# The fructose-bisphosphate, Aldolase A (ALDOA), facilitates DNA-PKcs and ATM kinase activity to regulate DNA double-strand break repair

**DOI:** 10.1038/s41598-023-41133-1

**Published:** 2023-09-13

**Authors:** Thais Sobanski, Amila Suraweera, Joshua T. Burgess, Iain Richard, Chee Man Cheong, Keyur Dave, Maddison Rose, Mark N. Adams, Kenneth J. O’Byrne, Derek J. Richard, Emma Bolderson

**Affiliations:** 1 Cancer and Ageing Research Program, Centre for Genomics and Personalised Health, Queensland University of Technology (QUT), Translational Research Institute (TRI), 37 Kent Street, Woolloongabba, Brisbane, Australia; 2 Princess Alexandra Hospital, Ipswich Road, Woolloongabba, Brisbane, QLD 4102 Australia

**Keywords:** Cell biology, DNA damage and repair

## Abstract

Glucose metabolism and DNA repair are fundamental cellular processes frequently dysregulated in cancer. In this study, we define a direct role for the glycolytic Aldolase A (ALDOA) protein in DNA double-strand break (DSB) repair. ALDOA is a fructose biphosphate Aldolase that catalyses fructose-1,6-bisphosphate to glyceraldehyde 3-phosphate (G3P) and dihydroxyacetone phosphate (DHAP), during glycolysis. Here, we show that upon DNA damage induced by ionising radiation (IR), ALDOA translocates from the cytoplasm into the nucleus, where it partially co-localises with the DNA DSB marker γ-H2AX. DNA damage was shown to be elevated in ALDOA-depleted cells prior to IR and following IR the damage was repaired more slowly. Consistent with this, cells depleted of ALDOA exhibited decreased DNA DSB repair via non-homologous end-joining and homologous recombination. In support of the defective repair observed in its absence, ALDOA was found to associate with the major DSB repair effector kinases, DNA-dependent Protein Kinase (DNA-PK) and Ataxia Telangiectasia Mutated (ATM) and their autophosphorylation was decreased when ALDOA was depleted. Together, these data establish a role for an essential metabolic protein, ALDOA in DNA DSB repair and suggests that targeting ALDOA may enable the concurrent targeting of cancer metabolism and DNA repair to induce tumour cell death.

## Introduction

Glucose metabolism and DNA double-strand break (DSB) repair pathways are two of the most frequently altered pathways identified in tumours^1,2^. The metabolic reprogramming that occurs in tumour cells involves a shift from predominantly oxidative phosphorylation to generate ATP, to aerobic glycolysis, is known as the Warburg effect^3^. The human Aldolase proteins are critical for glycolysis and are required to catalyse the reversible conversion of fructose-1,6-bisphosphate to glyceraldehyde 3-phosphate (G3P) and dihydroxyacetone phosphate (DHAP)^4^. In humans there are three isozymes, named Aldolase 1, 2 and 3, expressed at different levels in specific tissues during development^5^. Aldolase A (ALDOA) is predominantly found in adult muscle but is also ubiquitously expressed in most tissue types. Although the role of ALDOA in glycolysis has been best characterised, it has also been implicated in several other cellular processes including the regulation of muscle tissue, regulation of cell shape and motility, the organisation of the actin cytoskeleton and may also have a role in cell proliferation^6–11^. Significantly, ALDOA is upregulated in many tumour types and this suggests that its upregulation may contribute to the metabolic reprogramming observed in tumours^12–16^.

In addition to the reprogramming of cell metabolism, alterations in DNA repair gene expression are also frequently observed in various tumours types^17^. DNA repair pathways are critical for the maintenance of the genome and their downregulation can promote the accumulation of genomic errors that contribute to the rapid growth and adaptation observed in tumour cells. DNA double-strand breaks (DSBs) occur when both strands of the DNA duplex are broken and are generally regarded as the most deleterious lesion acquired by cells. DSBs can arise from exposure to exogenous agents, including ionising radiation (IR), but can also occur spontaneously during cellular processes, such as by reactive oxygen species produced during cell metabolism or by replication forks collapsing during replication. DSBs that are not repaired correctly can lead to gene mutations and chromosomal translocations, for this reason, defective repair of DSBs is considered a significant driver of tumourigenesis^18^. In eukaryotic cells, there are two major pathways for repairing double-strand DNA breaks: homologous recombination (HR) and non-homologous DNA end-joining (NHEJ), with NHEJ being the predominant method of DSB repair. As such, NHEJ functions during the entire cell cycle, while HR is activated in the late S and G2 phases of the cell cycle where there is a homologous sister chromatid available as a template in order to complete repair^19^. The main effector kinase that drives the NHEJ pathway is the catalytic subunit of the DNA-dependent kinase (DNA-PKcs) complex^20^. Homologous recombination can be mediated by either the Ataxia Telangiectasia Mutated (ATM) or Ataxia Telangiectasia and Rad3-related (ATR) kinases, depending upon whether the DSB is associated with replication forks^21^.

Associations between cellular metabolism and DNA repair pathways have long been hypothesised. Recent studies have shown that several proteins previously determined to have roles in cellular metabolism also have overlapping roles in DNA repair pathways (reviewed in^22,23^). In this study, we identify a novel association between cell metabolism and DNA repair pathways. Specifically, we show that in addition to its role in glycolysis, ALDOA also functions to regulate DNA DSB repair pathways via HR and NHEJ, likely through mediation of ATM and DNA-PK catalytic activity.

## Materials

### Reagents

#### Chemical reagents

All chemical reagents were purchased from Sigma unless otherwise stated.

#### Antibodies

The antibodies used were as follows: anti-ALDOA [HPA004177, Sigma-Aldrich, for western blot (WB) 1:1000 and 1:200 for immunofluorescence (IF)] anti-Flag M2 Antibody (F3165, Sigma-Aldrich, for WB 1:1000 and 1:300 for IF), anti-γ-Tubulin (T6557, Sigma-Aldrich, for WB 1:2000), anti-H3 (4499, Cell Signaling Technology, for WB 1:2000), anti-ATM (28739, Cell Signaling Technology, for WB 1:1000), anti-P-ATM S1981 (5883, Cell Signaling Technology, for WB 1:1000), Anti-P-ATM S1981 (ab36810, Abcam for IF: 1:200) anti-γH2AX (ab26350, Abcam, for WB and IF 1:1000), anti-p-DNA-PK S2056 (ab124918, Abcam, for WB 1:1000), anti-DNA-PKcs (12311, Cell Signaling Technology for WB 1:1000). Fluorescent secondary antibodies used: Donkey anti-Mouse 800 nm (LiCor; IRDye 800CW 926-32212, 1:5000 for WB), Donkey anti-Rabbit (LiCor; IRDye 680LT 926-28023, 1:5000 for WB) and Alexa Fluor 488 (Cat# A32766, Molecular Probes, 1:200 for IF) and 594 (Cat# A32754, Molecular Probes, for IF 1:200).

### Biological resources

#### Cell lines

The U2OS and HEK293T cells were obtained from CellBank Australia (catalogue numbers 92022711 and 85120602, respectively). U2OS cells were grown in RPMI media (Life Technologies), supplemented with 10% FCS (Thermo Fisher Scientific). HEK293T cells were grown in DMEM media (Life Technologies), supplemented with 10% FCS (Thermo Fisher Scientific). Cell lines were grown at 37 °C, 5% CO_2_ and at atmospheric O_2_, unless otherwise stated.

#### Constructs

The Flag-ALDOA construct was synthesised by Genscript in pcDNA3.1 + N-DYK vector in the BamHI-XhoI cloning sites. These constructs were sequenced using the CMV primer (5′-CGCAAATGGGCGGTAGGCGTG-3′). ALDOA overexpression was analysed 24 h post-transfection. All DNA constructs were transfected using Fugene HD (Promega), as per manufacturer’s instructions.

#### siRNA

Depletion of ALDOA was performed using the mission siRNA transfection (Sigma-Aldrich) the sequences ALDOA si 1 (GUGUCAUCCUCUUCCAUGA [dT]), ALDOA si 2 (CUGUCACUGGGAUCACCUU [dT] and ALDOA si 3 (GUGUCAUCCUCUUCCAUGA [dT]). Optimal depletion of ALDOA was obtained with a final concentration of 20 nM siRNA (Sigma), and transfections with RNAimax (Life technologies) were performed as per manufacturer’s instructions. Cells were typically assayed 96 h after transfection.

## Methods

### Immunoblotting

Cells were lysed as previously^24^ (lysis buffer: 20 mM HEPES pH7.5, 250 mM KCl, 5% glycerol, 10 mM MgCl_2_, 0.5% Triton X-100, Protease inhibitor cocktail (Roche) and phosphatase inhibitor cocktail (Cell Signaling)) and sonicated. Lysates were cleared by centrifugation. Typically, 20 μg of protein lysate was separated on a Bolt 4–12%, Bis–Tris, 1.0 mm, Mini Protein Gel (Invitrogen) blocked in Odyssey buffer (LiCor Biosciences) and immunoblotted with the indicated antibodies. Immunoblots were imaged using an Odyssey infrared imaging system (LiCor).

### Immunoprecipitation

Cells were treated as stated then lysed using Pierce lysis buffer (25 mM Tris HCl pH 7.4, 150 mM NaCl, 1% NP-40, 1 mM EDTA, 5% glycerol, plus Pierce Universal nuclease (as per manufacturer’s instructions)), and 800–1000 μg of protein was used for each immunoprecipitation reaction. The lysate was incubated with the desired antibody overnight at 4 °C with gentle agitation. The antibody–antigen complex was incubated with protein A or protein G Dynabeads (Thermofisher) for 1 h at 4 °C. Following capture, beads are washed 5× with Pierce lysis buffer, then the proteins were eluted by heating to 90 °C in SDS loading dye (10% SDS, 500 mM DTT, 50% Glycerol, 250 mM Tris–HCL and 0.5% bromophenol blue dye, PH 6.8).

### Immunofluorescence

Cells were seeded the day before siRNA transfection. Following siRNA transfection cells were allowed to grow for 72 h before treatment or mock-treatment with the indicated DNA damaging agent. After treatment cells were treated with an extraction buffer (20 mM Hepes, 20 mM NaCl, 5 mM MgCl_2_, 0.5% IGEPAL), to remove soluble proteins to enable study of chromatin-bound proteins^25^ for 5 min before fixation in 4% paraformaldehyde (PFA) for 15 min at room temperature. For non-extracted immunofluorescence experiments this initial step was not applied, and the cells were direct fixed with 4% (PFA). Cells were permeabilised with 0.2% Triton X-100 for 5 min and blocked in 3% BSA for 30 min. Cells were incubated with indicated primary antibodies and Alexa-conjugated secondary antibodies for 1 h each at room temperature. In order to visualise the colocalisation between ALDOA and DNA-PK, and ALDOA and ATM, cells were treated as above but with the addition of 0.3 mg/ml RNase A to the extraction buffer^26^. Cells were stained with Hoechst 33342, before imaging on a Delta Vision PDV microscope, 60×/1.42 or 100×/1.42 Oil objective (Applied Precision, Inc). All immunofluorescence figures were assembled using ImageJ. High content imaging was performed using the InCell Analyzer 6500 Imaging System (GE Healthcare Life Sciences). Nuclear staining intensity was analysed using the InCell Incarta software (GE Healthcare Life Sciences) with a minimum of 500 nuclei quantified per each independent experiment and the results shown represent the mean and S.D. of 3 independent experiments. The % of protein co-localization distributed across the cell was analyzing using Image J. The analysis between the green and red channels coefficient values was performed using softWoRx software (Applied Precision, Issaquah WA) on a representative area. For the analysis 15 cells were used for each condition from 3 independent experiment.

### CellTiter-Glo cell viability assay

U2OS cells were transfected with control or ALDOA siRNA as previously. 48 h post-transfection, 4000 cells were plated into wells of a 96-well plate (Corning). Cells were mock-treated or treated with the indicated doses of camptothecin (Sigma-Aldrich) or hydrogen peroxide (Sigma-Aldrich) as shown and incubated for 72 h. CellTiter-Glo 2.0 (Promega Corporation) was added to each well and luminescence was measured using a PHERAstar FSX detection system (BMG Labtech) and normalised back to the untreated wells^27^.

### Apoptosis assay

U2OS cells were transfected with control or ALDOA siRNA as previously. Cell death was quantified using an Annexin V-FITC apoptosis detection kit (Enzo Life Sciences, ALX-850-020-KI02), as per manufacturer’s instructions. Cells death was assayed using a CytoFLEX Flow Cytometer (Beckman Coulter Life Sciences) and data was analysed using FlowJo analysis software.

### Subcellular fractionation

Subcellular fractions were isolated using a Thermo Scientific Subcellular Fractionation Kit (ThermoFisher) as per the manufacturer's instructions^28^. Fractions were immunoblotted using the indicated antibodies, including antibodies against control proteins for each fraction.

### Homologous recombination and non-homologous end-joining assays

For the integrated homologous recombination and non-homologous end-joining assays; the pLCN DSB Repair Reporter (DRR) (Addgene plasmid #98895; http://n2t.net/addgene:98895; RRID: Addgene_98895) and pCAGGS DRR mCherry Donor EF1a BFP were a gift from Jan Karlseder (Addgene plasmid #98896; http://n2t.net/addgene:98896; RRID: Addgene_98896). Stable U2OS cells containing the pLCN DSB Repair Reporter were generated, and the assay carried out as in^29^. Briefly, the stable U2OS cells were transfected with the indicated siRNAs 48 h before transfection with *Isce1* and an exogenous donor for HR (pCAGGS DRR mCherry Donor EF1a BFP). 48 h after transfection cells were processed for FACS to detect BFP (as a control for transfection efficiency) GFP or mCherry expression. Repair by NHEJ or HR leads to GFP or mCherry expression, respectively.

### Comet assay

The neutral comet assay was employed to measure DSBs following IR treatment, as performed previously^30^. Briefly, U2OS cells were treated or mock-treated with IR (6 Gy). Immediately after IR, 1 h, and 4-h post-IR, cells were embedded in agarose, lysed and subjected to electrophoresis, according to the Trevigen neutral comet assay protocol, with a few variations. The lysis buffer used contained: 2.5 M NaCl, 100 mM EDTA, 10 mM Tris (pH 10), 1% Triton X-100. The electrophoresis was carried out with TBE buffer. Single cells were stained with Sybr Green I (Invitrogen) and at least 100 randomly selected cells per condition were analysed using imageJ software and the Olive tail moment was calculated^31^.

### Colony-forming assay

U2OS cells were transfected with control or ALDOA siRNA. 48 h after transfection 700 cells were seeded into wells of a 6-well plates. Cells were treated or mock-treated with the 0–6 Gy IR and incubated for 10 days. Colonies were stained with 4% methylene blue in methanol, manually counted and normalised back to the number of colonies in the untreated wells^32^.

### Single shot quantitative proteomics (adapted from^25,33^)

U2OS cells were transfected with control or ALDOA siRNA and prepared as per the siRNA protocol. The dried tryptic peptides were resuspended in 2% (v/v) acetonitrile containing 1% (v/v) TFA and analysed using ThermoFisher Scientific Ultimate 3000 RSLC nano ultra high pressure liquid chromatography system (nUHPLC) interfaced to hybrid quadrupole-Orbitrap Q Exactive Plus mass spectrometer. Acidified peptides were loaded onto Acclaim Pepmap trap (300 µm I.D. × 5 mm, C18, 5um particle size, 100 Å) at 10 µl/min in 98% solvent A (0.1% (v/v) formic acid) and 2% solvent B (80% (v/v) acetonitrile, 0.1% (v/v) formic acid) for 3 min and the peptides were subsequently separated with a 140 min multi-step gradient using a pre-equilibrated analytical column (ThermoFisher Scientific, Easy-Spray C18, 75 µm I.D. × 500 mm, 2 µm particle size, 100 Å ) at a flow-rate of 0.25 µl/min and temperature of 40 °C. The Q Exactive Plus was equipped with a Easy-Spray ion source (Thermo Fisher Scientific) containing integrated temperature control module and glass emitter. Spray voltages were between 1.5 and 1.9 kV and no sheath, sweep or auxiliary gases were used. The Q Exactive Plus was operated in a data-dependent mode to automatically switch between Orbitrap-MS and Orbitrap-MS/MS acquisition. After accumulation to a target value of 1,000,000 charges in the Orbitrap at a maximum injection time of 50 ms, survey full scan MS spectra (from m/z 350–1400) were acquired in the Orbitrap with resolution r 70,000 at m/z 400. Depending on the signal intensity, up to 10 most intense ions were sequentially isolated, fragmented in the HCD cell and recorded in the orbitrap after accumulation to a target value of 50,000 and a resolution of 17,500 at a maximum injection time of 50 ms. For accurate mass measurements the lock mass option was enabled in MS mode and the polydimethylcyclosiloxane ions (protonated Si(CH3)2O6; m/z 445.120025 from ambient air) were used for real time internal recalibration. General mass spectrometric conditions were: ion transfer tube temperature, 285 °C; repeat count, 1; exclusion duration, 90 s; isolation window, 1.4 m/z; peptide match, preferred; and spectrum data type, profile. Protein identification and label-free quantification were performed using MaxQuant (*ver 1.6.10.1*) ^34^ MaxQuant was used to extract peak lists from the Xcalibur *.raw* files (ThermoFisher Scientific, Germany) and the embedded database search engine Andromeda^35^ as used to assign peptide sequences to the fragmentation spectra. The database searched consisted of the reference proteome for Homo Sapiens *(20,369 canonical non-redundant sequences downloaded from *www.uniprot.org* on 5th September 2019)*, reversed sequences and the MaxQuant contaminant database. For protein identification, the PSM and protein FDRs were set to 0.01 and both unique and razor peptides were used for label-free quantification. For this dataset a minimum of 2 peptides were required for identification of a protein. Protein quantification was performed using the LFQ intensities reported by MaxQuant. After left-censored imputation strategy, a moderated t-test was applied using limma^36,37^ as applied to control the FDR.

### Statistical analyses

Statistical analysis of the results was made using GraphPad Prism software. T-test (two-tailed) were used for statistical analysis, unless otherwise stated. Data are presented as means and standard deviation (SD) from ≥ 3 independent experiments (unless otherwise stated). Statistical significance is represented by *P value < 0.05; **P value < 0.01; ***P value < 0.001; ****P value <  0.0001.

### Ethical approval

Experimental procedures using human cell lines were approved by the Queensland University of Technology; Human Research Ethics Committee (approval number 1900000269). All methods were performed in accordance with the relevant guidelines and regulations of Queensland University of Technology, according to the Australian Code of the Responsible Conduct of Research.

## Results

### Depletion of ALDOA leads to changes in DSB repair pathways

**Figure 1:**
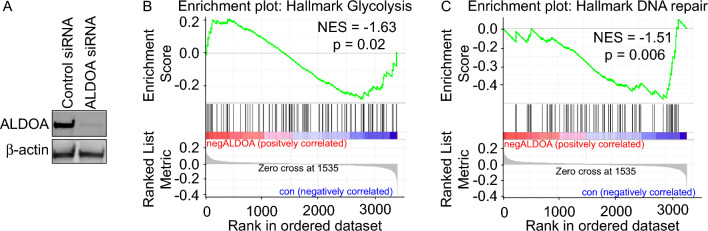
ALDOA depletion leads to changes in glycolysis and DNA repair pathways. (**A**) U2OS cells were transfected with control siRNA or ALDOA siRNA sequences. 96 h following transfection whole cell lysates were prepared and immunoblotted with the indicated antibodies. (**B**,**C**) Cells were treated as in a, followed by LC/MS–MS and gene set enrichment analysis (GSEA) was performed with the canonical pathway, or biological process, gene sets in GSEA Molecular Signatures Database. The green curve corresponds to the ES (enrichment score) curve, which is the running sum of the weighted enrichment score obtained from GSEA software, while the normalised enrichment score (NES) and the corresponding P value are reported within each graph (**B**) ALDOA depletion downregulates glycolysis pathways. (**C**) ALDOA depletion leads to the downregulation of DNA repair pathways.

In order to identify other potential roles for the glycolytic protein Aldolase A (ALDOA), we firstly confirmed the depletion of ALDOA from U2OS cells using siRNA (Fig. 1A). Quantitative mass spectrometry was then carried out to compare peptide levels from control cells and cells depleted of ALDOA. GSEA analysis was used to identify differentially regulated pathways and showed that depletion of ALDOA led to a significant decrease in glycolysis pathways, as anticipated (Fig. 1B). Unexpectedly, we also observed significant changes in the peptide levels of DNA DSB repair proteins in ALDOA depleted cells, suggesting that ALDOA could have a potential role in regulating DNA repair (Fig. 1C). Further analysis of DNA repair protein pathways implicated changes in the peptide levels of proteins involved in DNA double-strand break repair pathway signatures, including Histones, DNA-modifying enzymes and splicing factors (Supplementary Fig. 1).

### ALDOA responds to DNA damage

**Figure 2:**
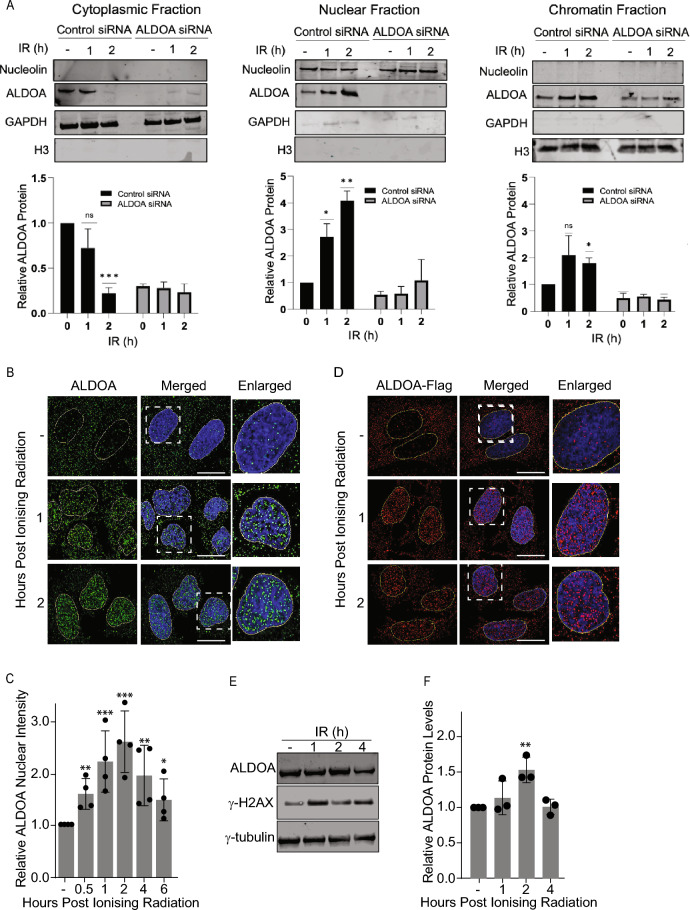
ALDOA responds to IR-induced DNA damage. (**A**) ALDOA migrates from the cytoplasm to the nucleus and chromatin following ionising radiation. U2OS cells were treated with 6 Gy IR, and processed into cellular fractions, at the indicated timepoints. Lysates were immunoblotted with the indicated antibodies. GAPDH is included as cytoplasmic control, Nucleolin as a nuclear control and Histone H3 as a chromatin control. The histograms show the relative ALDOA protein levels normalized to the control proteins for each fraction. (**B**) ALDOA migrates from the cytoplasm to the nucleus following ionising radiation. U2OS cells were treated with 6 Gy IR, pre-extracted and fixed, at the indicated timepoints. Cells were stained with the indicated antibodies and imaged. (**C**) Cells were treated as in b. The histograms represent nuclear intensity of endogenous ALDOA protein in cells. (**D**) U2OS cells were transfected with Flag-ALDOA, treated with 6 Gy IR, and pre-extracted and fixed, at the indicated timepoints. Cells were stained with ALDOA antibodies and imaged. (**E**) ALDOA protein levels increase following IR treatment. U2OS cells were treated with 6 Gy IR and whole cell lysates prepared at the indicated times post-IR. Lysates were immunoblotted with the indicated antibodies. γ-H2AX was used as a marker for DNA damage induction. (**F**) The immunoblots from e, were analysed via densitometry. The histogram shows ALDOA protein levels at each timepoint normalised to β-actin levels. Data points represent the mean and error bars represent the S.D. of three independent experiments, *P < 0.05 **P < 0.01, ***P < 0.001. Immunofluorescence scale bars represent 10 μm.

To identify whether ALDOA had a role in the repair of DNA damage, we next determined whether ALDOA protein responded to DNA damage, induced by ionising radiation (IR). Using cellular fractionation, in the absence of DNA damage, consistent with previous reports^12^ ALDOA was observed to be primarily localised in the cytoplasm (Fig. 2A). However, within 1 and 2 h treatment of cells with 6 Gy IR, levels of ALDOA were observed to decrease in the cytoplasm and increase in both the nuclear and chromatin fractions. Supporting this observation, in immunofluorescence studies both endogenous ALDOA (Fig. 2B,C) and exogenously expressed Flag-ALDOA (Fig. 2D, Supplementary Fig. 2) were observed to decrease in the cytoplasm and increase in the nucleus. Taken together, this suggests that ALDOA translocates from the cytoplasm to the nucleus and chromatin in response to DSBs.

To further characterise ALDOA function in the response to DNA damage we next examined ALDOA protein levels via immunoblot and mRNA levels via quantitative PCR. Total ALDOA protein levels (Fig. 2E,F) and ALDOA mRNA levels (Supplementary Fig. 3) were observed to increase 2 h after IR, supporting the notion that ALDOA is upregulated in response to DNA DSBs.

### ALDOA is required for DSB repair

**Figure 3:**
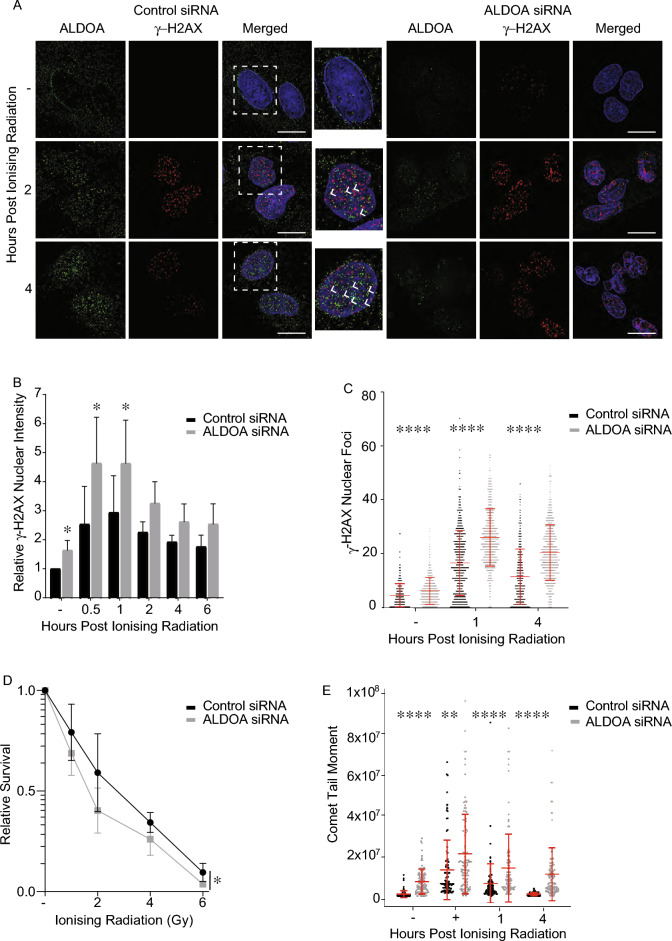
ALDOA is required for DNA repair. (**A**) ALDOA partially localises with γ-H2AX following ionising radiation in U2OS cells. Representative cells stained with the indicated antibodies at the indicated times post 6 Gy IR are shown. (**B**) The relative nuclear γ-H2AX intensity detected by immunofluorescence in control or ALDOA siRNA transfected cells. (**C**) The number of γ-H2AX foci per cell at the indicated times post-IR in control and ALDOA siRNA transfected cells. Data points represent the γ-H2AX foci/nuclei from a minimum of 250 nuclei. (**D**) ALDOA depletion induces radiosensitivity in U2OS cells. U2OS cells were transfected with control and ALDOA siRNA. 48 h after transfection cells were seeded into 6-well plates. Cells were treated or mock-treated with the indicated doses of IR and colonies were stained and counted 10 days after exposure to IR. Data shown represents the mean and SD of two independent experiments and the statistical analysis was via two-way ANOVA. (**E**) Depletion of ALDOA causes delayed repair of IR-induced DNA damage. Data points represent the Olive tail moment from a minimum of 100 cells. + IR represents immediately following IR treatment. Data shown is the mean and SD of two independent experiments. Unless otherwise stated, data points represent the mean and error bars represent the SD of three independent experiments and t-test was used for statistical analysis: *P < 0.05, ***P < 0.001, ****P < 0.0001. Immunofluorescence scale bars represent 10 μm.

Since it was observed from both immunofluorescence and cell fractionation studies that ALDOA translocated from the cytoplasm into the nuclear and chromatin fractions, we next determined whether ALDOA was directly recruited to chromatin proximal to DNA DSBs, using immunofluorescence on irradiated cells. One of the earliest responses to DNA DSBs is the phosphorylation of H2AX on serine 139 (known as γ-H2AX after phosphorylation) in the regions of chromatin proximal to the break, enabling it to be used as a marker of DSBs^38^. In cells treated with 6 Gy IR, ALDOA could be observed to partially overlap with 38% of γ-H2AX foci, the Pearson coefficient r value was 0.36 2 h post IR suggesting it may at least in part be recruited to the chromatin proximal to a break (Fig. 3A). To further determine the role of ALDOA in the repair of DNA DSBs induced by IR, we next analysed γ-H2AX foci resolution, as a method to establish the kinetics of DNA DSB repair in the absence of ALDOA. We found that cells depleted of ALDOA showed higher γ-H2AX levels than control cells both before and after IR treatment, as measured by nuclear intensity (Fig. 3B) and number of γ-H2AX foci (Fig. 3C). This was also confirmed with alternative ALDOA siRNA sequences (Supplementary Fig. 4A,B). The increased γ-H2AX levels were also observed in ALDOA-depleted cells via immunoblotting (Supplementary Fig. 4C) Conversely, we found that expression of an siRNA resistant wild-type ALDOA plasmid rescued the γ-H2AX levels, restoring them to below those observed in control cells, potentially implicating ALDOA in facilitating DNA DSB repair (Supplementary Fig. 5). This suggests that in the absence of ALDOA, DSBs, as measured by γ-H2AX may accumulate in the absence of exogenous damage and that once DSBs are induced by IR these breaks are also repaired more slowly.

In order to determine whether ALDOA is required for cell survival following induction of DNA damage by IR we next carried out clonogenic assays in cells depleted of ALDOA. Here, we saw a decrease in the colony-forming ability of ALDOA-depleted cells compared to control cells, following IR treatment (Fig. 3D). This suggests that ALDOA is required for cell survival following IR-induced DNA damage and supports the mass spectrometry data implicating ALDOA in the repair of DSBs. Also supporting a role for ALDOA in double-strand break repair, ALDOA-depleted cells were also sensitive to the topoisomerase I inhibitor, camptothecin (CPT), that causes single-strand breaks which are processed into double-strand breaks in S-phase^39^ (Supplementary Fig. 6a). ALDOA-depleted cells were also sensitive to hydrogen peroxide, which causes oxidative damage to DNA, which is repaired by the base excision repair pathway (Supplementary Fig. 6b). This suggests that ALDOA may also have direct or indirect roles in other DNA repair pathways, in addition to double-strand break repair.

To confirm that the increase in γ-H2AX levels corresponds to an increase in DSBs and not a change in γ-H2AX regulation we next carried out neutral comet assays. Here, it was found that there was a delay in repairing DSBs in ALDOA-deficient cells compared to control cells. We also found that ALDOA-depleted cells had significantly longer length comet tail moments than control cells in both untreated cells and after IR at all timepoints analysed, supporting the observation of increased γ-H2AX levels (Fig. 3E, Supplementary Fig. 7).

### ALDOA is required for DSB repair via NHEJ and HR

**Figure 4:**
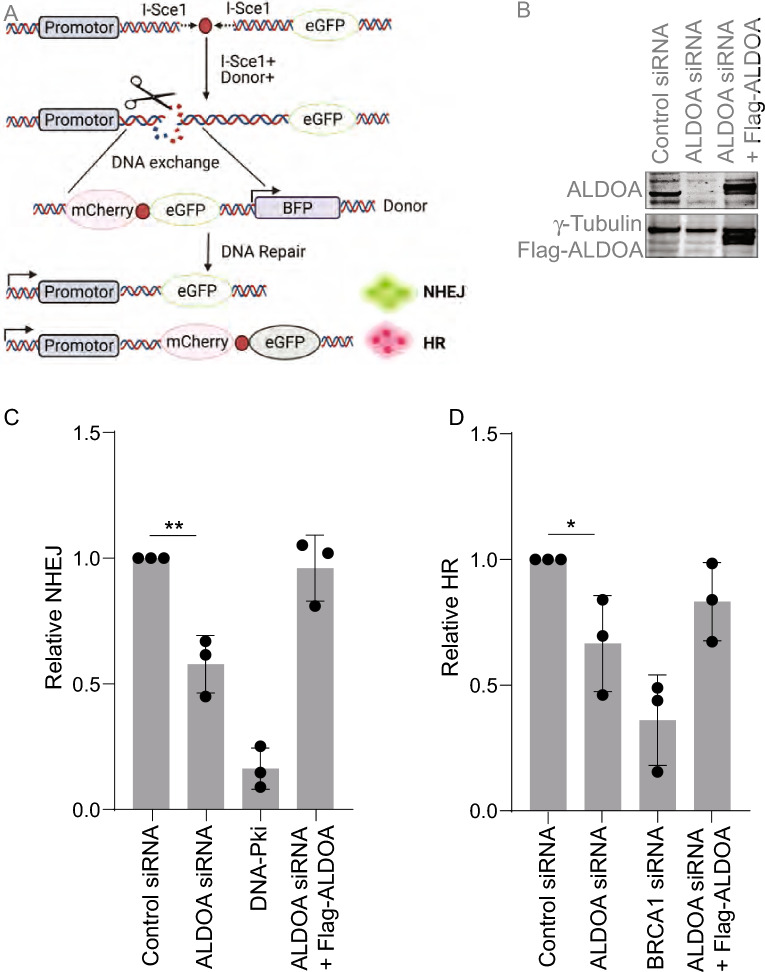
ALDOA is required for DNA double-strand break repair. (**A**) Schematic of the DSB repair reporter assay used, repair by NHEJ or HR leads to GFP or mCherry expression, respectively^29^ Repair of DSBs by NHEJ and HR were assayed using the U2OS DSB.3 U2OS cell line-based chromosomal reporter system, containing a single integrated GFP reporter with two I-SceI target sites in inverted orientation, flanking an out of frame ATG upstream of the GFP reporter. Cleavage by I-SceI removes the ATG, leaving non-compatible ends. Figure generated with Biorender.com. (**B**) U2OS cells containing the stably integrated HR/NHEJ reporter construct were depleted of ALDOA using siRNA and transfected with Flag or Flag-ALDOA, confirmed via immunoblot. (**C**,**D**) Depletion of ALDOA results in decreased NHEJ (**C**) and HR (**D**). Cells were transfected with control or ALDOA siRNA, then transfected with pcDNA3.1/I-SceI after 48 h. Cells transfected with empty Flag or siRNA resistant Flag-ALDOA showed the restoration of HR/NHEJ activity. Unless otherwise stated, data points represent the mean and the error bars represent the SD of three independent experiments and t-test was used for statistical analysis: *P < 0.05 **P < 0.01.

Since ALDOA responded to induction of DSBs and depletion of ALDOA led to defective repair of DSBs, we next wanted to determine which type of DSB repair ALDOA was involved in. To address this, we next analysed the efficiency of DSB repair via HR and NHEJ in ALDOA-depleted cells, using previously described cell reporter assays (Fig. 4A)^29,40^. We found that depletion of ALDOA led to a significant decrease in the NHEJ frequency compared to control cells (Fig. 4B,C). In addition, we also observed a significant decrease in the HR frequency in ALDOA-depleted cells (Fig. 4D). Significantly, expression of an siRNA resistant wild-type ALDOA plasmid rescued both the NHEJ and HR levels to similar levels to control cells (Fig. 4B–D). Taken together these data suggest that ALDOA is required for major DSB repair pathways. Cell cycle analysis indicated a decrease in S-phase cells and increase in G2 cells in ALDOA-depleted cells (Supplementary Fig. 8A,B). Since both NHEJ and HR are defective in the absence of ALDOA, this likely represents an authentic DSB repair defect, rather than a defect induced by changes in cell cycle distribution. We also determined that ALDOA-depletion did not have an effect on cell proliferation, using Incucyte analysis, at the 48–72 h post-siRNA transfection timepoint when the DNA repair assays were carried out (Supplementary Fig. 9a). However, it was observed that with prolonged ALODA-depletion, the cellular proliferation did significantly slow down (Supplementary Fig. 9b). ALDOA-depletion did not lead to significantly more apoptosis than control transfected cells (Supplementary Fig. 10), suggesting that apoptosis was not a factor in the DNA repair assays.

### ALDOA interacts with DNA-PKcs and ATM complexes

**Figure 5:**
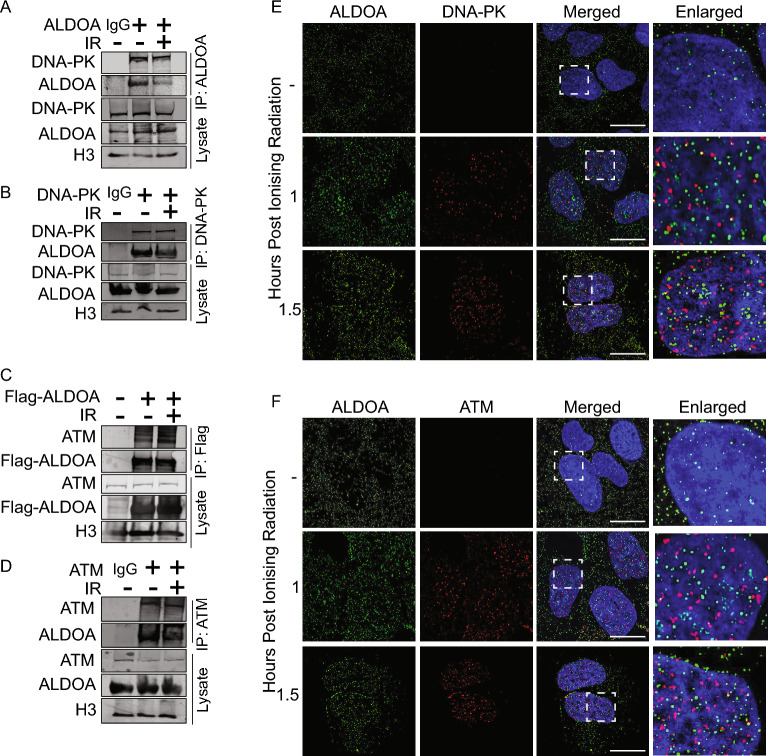
ALDOA interacts with DNA-PK- and ATM-containing complexes. (**A**) 293 T cells were exposed to 6 Gy IR and lysates were prepared for immunoprecipitation with ALDOA antibodies at the indicated timepoints. Eluted proteins and whole cell lysates were separated by electrophoresis and immunoblotted with antibodies against ALDOA and DNA-PK. (**B**) The reciprocal immunoprecipitation with an anti-DNA-PK antibody was also able to detect an interaction with ALDOA. (**C**) 293 T cells were transfected with plasmids encoding Flag-ALDOA, 24 h prior to cell lysis and immunoprecipitation with ALDOA antibodies. Associating proteins were immunoprecipitated from 293 T whole cell lysates prepared from cells that had been either left untreated or exposed to 6 Gy IR and harvested after the indicated IR time point. Eluted proteins and whole-cell lysates were separated by electrophoresis and immunoblotted with antibodies against ALDOA and ATM. (**D**) The reciprocal immunoprecipitation with anti-ATM antibodies was also able to detect an interaction with ALDOA. (**E**) ALDOA and DNA-PKcs partially colocalise following ionising radiation. U2OS cells were treated or mock-treated with 6 Gy IR and treated with extraction buffer (+ RNase) prior to fixation in PFA at the indicated timepoints. Cells were then stained with the indicated antibodies. (**F**) ALDOA and P-ATM (S1981) partially colocalise following ionising radiation. U2OS cells were treated or mock-treated with 6 Gy IR and treated with extraction buffer prior to fixation in PFA at the indicated timepoints. Cells were then stained with the indicated antibodies. Unless otherwise stated, data shown are representative of three independent experiments. Immunofluorescence scale bars represent 10 μm.

A previous study has suggested that ALDOA may have a role in the regulating DNA-PKcs, through an interaction which controls p53 phosphorylation^41^. Since DNA-PKcs is essential for DSB repair via NHEJ, we next examined whether we could detect an interaction between ALDOA and DNA-PKcs. Immunoprecipitations using ALDOA antibodies showed an association between DNA-PKcs and ALDOA pre- and post-IR treatment (Fig. 5A). Conversely, immunoprecipitations using DNA-PKcs antibodies were performed, and an association was also detected with ALDOA using this method (Fig. 5B). Since depletion also disrupted HR, we reasoned that ALDOA may also interact with other kinases involved in DNA repair. The Ataxia Telangiectasia Mutated (ATM) and Ataxia Telangiectasia and Rad3-Related (ATR) kinases are required for HR, so we next examined whether ALDOA interacted with ATM. Immunoprecipitations using Flag antibodies, showed an association between ATM and Flag-ALDOA pre-IR treatment, which increased modestly after IR (Fig. 5C). Immunoprecipitations using ATM antibodies also showed an association between ATM and ALDOA before IR treatment, which increased within 1 h of IR (Fig. 5D). Under our experimental conditions we were unable to detect any association between ATR and ALDOA (Supplementary Fig. 11). Taken together this shows that ALDOA interacts with DNA-PKcs and ATM, either directly or through another component of the complex. Since DNA-PK and ATM directly mediate the repair of DSBs and depletion of ALDOA leads to a DSB repair defect, this raises the question that the presence of ALDOA may promote the activity of these kinases to facilitate DSB repair.

To further address the role of ALDOA in DSB repair we next examined whether ALDOA localises to similar regions to DNA-PKcs and ATM following IR. In order to determine whether ALDOA and DNA-PKcs colocalised after DNA damage, we treated cells with an extraction buffer plus RNase to digest RNA, allowing visualisation of chromatin-associated DNA-PKcs following IR, as reported previously^26^. Using this method, we were able to detect that ALDOA foci overlapped with 16% and 36% of DNA-PKcs foci at 1 h and 1.5 h, respectively, following 6 Gy IR (Fig. 5e). Similarly, ALDOA was also observed to colocalise with ATM phosphorylated on S1981, suggested to be activated ATM, 11% and 23% of ALDOA foci and ATM S1981 foci overlapped 1 h and 1.5 h post-IR treatment, respectively (Fig. 5f).

### ALDOA regulates DNA-PKcs and ATM activity

**Figure 6:**
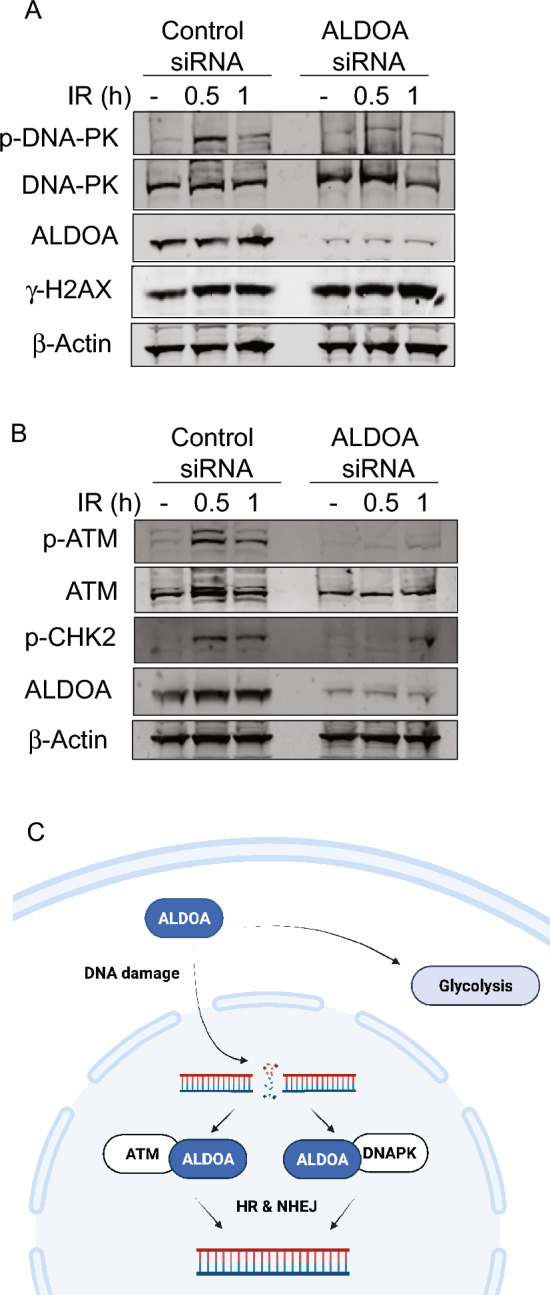
ALDOA depletion significantly decreases the autophosphorylation of ATM and DNA-PKcs. (**A**,**B**) U2OS cells were treated with 6 Gy of IR and lysates prepared at the time points indicated. Immunoblots were incubated with the indicated antibodies. (**C**) The schematic shows a proposed mechanism of ALDOA-dependent regulation of DNA DSB repair. Following IR-induced DNA damage ALDOA translocates from the cytoplasm to the nucleus, where it forms a complex with ATM and/or DNA-PK promoting their kinase activity, leading to repair of the DSB. Figure generated with Biorender.com.

The interaction and partial colocalisation of ALDOA with ATM and DNA-PKcs complexes, together with the defects in HR and NHEJ, implicated ALDOA in regulating their activity therefore we next examined the ATM- and DNA-PKcs IR-dependent induced signalling pathways. We observed that when ALDOA was depleted there was a significant decrease of the auto-phosphorylation of ATM and DNA-PKcs, together with a decrease in the phosphorylation of a downstream substrate of ATM, Chk2, suggesting that ATM and DNA-PKcs activity is disrupted in the absence of ALDOA (Fig. 6A,B).

Taken together these data support a model whereby ALDOA regulates the kinase activity of ATM and DNA-PKcs and subsequently mediates the two major DSB repair pathways, HR and NHEJ (Fig. 6C), providing evidence of a direct association between metabolic processes and DNA repair.

## Discussion

Here, we establish a role for ALDOA in the DNA damage response through the regulation of the key DNA damage repair kinases DNA-PKcs and ATM. Initial mass spectrometry analysis of ALDOA depleted cells, implicated ALDOA in regulation of DNA DSB repair proteins. Supporting a role in DNA repair, following IR ALDOA was observed to rapidly translocate from the cytoplasm to the nucleus and chromatin fractions, where it displays some partial colocalization with γ-H2AX, ATM and DNA-PK. In order to confirm that ALDOA localises to the sites of DSBs, further analysis is required, but our data suggest that this is a possibility. Depletion of ALDOA led to an increase in γ-H2AX foci both prior to and after IR treatment, which, was consistent with increased DNA damage, as measured by a neutral comet assay. ATM and DNA-PKcs are known to phosphorylate H2AX and it may be expected that we would observe a decrease in IR-induced γ-H2AX in ALDOA-depleted cells that exhibit defective ATM and DNA-PKcs activity. However, ATR, VRK1 and the metabolic protein PKM2 have also been shown to have the ability to phosphorylate H2AX on serine 139. It is possible that the activity observed may be due to upregulation of these other kinases in the absence of ALDOA protein, with the slower repair of DSBs leading to the slower resolution of γ-H2AX foci^42–44^. ALDOA deficient cells also retain over 40% of their NHEJ activity suggesting that some DNA-PKcs activity remains, which may be sufficient to phosphorylate H2AX.

Consistent with the increased level of DSBs observed in ALDOA-depleted cells cellular sensitivity to IR, ALDOA was also shown to be required for efficient DSB repair via HR and NHEJ. Providing an explanation for this, ALDOA was also found to interact with and promote the activity of the effector kinases of NHEJ and HR, DNA-PKcs and ATM, respectively. This observation supports a previous study which showed that ALDOA interacts with DNA-PKcs and enhances its kinase activity leading to increased phosphorylation of p53, a DNA-PK substrate^41^. Supporting this, another study also showed that ALDOA interacts with DNA-PK to regulate glycolysis and that inhibition or depletion of DNA-PK led to a decrease in aldolase activity in castrate-resistant prostate cancer cells^45^. We and others have shown that ALDOA regulates DNA-PK activity and, together with evidence that DNA-PK also regulates ALDOA activity, this supports the potential existence of a feedback loop between the two proteins, however further investigation is required to determine this. Together with the data shown here this supports the hypothesis that the ALDOA-DNA-PK interaction promotes the activity of both enzymes to facilitate both DNA repair and glycolysis. Further research is required to determine whether this applies only in disease states, such as cancer, or also under normal cellular conditions.

The process by which ALDOA may promote the activity of ATM and DNA-PKcs is currently unknown but we speculate it may be that the binding of ALDOA leads to a conformational change in the catalytic domains of DNA-PK and ATM, enhancing their kinase activity. ATM has been shown to exist as an inactive dimer or higher order multimer prior to IR. Following DNA damage intermolecular autophosphorylation occurs, leading to rapid dissociation of the ATM dimers and an increase in ATM catalytic activity^46^. In the case of ATM, it may be that the binding of ALDOA, directly to ATM or to an ATM-associated complex, may promote the destabilization of the ATM dimer and promote the autophosphorylation and subsequent activation of each ATM molecule. Although ALDOA has not been implicated in regulating ATM activity previously, in contrast to our data, a previous study found that overexpression of ALDOA led to downregulation of ATM levels in pancreatic cancer cells, suggesting that ALDOA levels need to be tightly regulated to avoid disruption of DNA repair pathways^47^.

In contrast to the homodimer ATM, the DNA-PK complex consists of the DNA-PKcs, along with two smaller DNA-binding proteins, Ku70 and Ku80. Several mechanisms of DNA-PKcs regulation have been highlighted previously, including regulation of its interaction with DNA ends by Ku70/80, which is required for its activation and also its auto-phosphorylation^48^. It can therefore be suggested that the binding of ALDOA may induce a conformational change that may stabilise the interaction between DNA-PKcs and Ku70/80 to increase the activity of the whole complex. Further investigation is required to determine the precise mechanism of this ALDOA-dependent regulatory mechanism of ATM and DNA-PK activity. However, given that ALDOA-depleted cells only show a slight increase in IR sensitivity and retain some HR and NHEJ activity, this supports the assumption that both ATM and DNA-PK maintain some catalytic activity in the absence of ALDOA.

The observed ALDOA-dependent down-regulation of DNA repair proteins in the mass spectrometry analysis could be at the transcriptional or protein level and it is likely that the alterations in the levels of multiple proteins in DNA repair pathways may, at least in part, contribute towards the decreased DNA repair and subsequent increased γ-H2AX signaling observed in ALDOA-depleted cells. However, further study is required to identify the mechanisms behind this. In addition to this potential role in regulating the expression or stability of DNA repair proteins, our data suggest that ALDOA also has a direct role in DNA repair.

ALDOA is not the first protein identified to have dual roles in metabolism and DNA repair pathways. A direct role in DNA repair has also been identified for the Phosphoglycerate mutase 1 (PGAM1), a key glycolytic enzyme that has been shown to coordinate metabolic process including glycolysis, PPP and serine biosynthesis. Specifically, PGAM1 was shown to have a role in promoting DSB repair via HR through the regulation of CtIP stability^49^. In addition, Pyruvate Kinase M2 (PKM2) is an enzyme that converts phosphoenolpyruvate and ADP into pyruvate to generate ATP, essential for glucose homeostasis. Similarly to ALDOA, PKM2 was shown to translocate from the cytosol to the nucleus following DNA damage^44^. A consequence of this migration was that PKM2 could directly phosphorylate H2AX on serine 139. PKM2 was also shown to be phosphorylated by ATM and this modification was shown to be required for the recruitment of CtIP to sites of DNA damage and subsequent HR^50^. The identification of ALDOA, a protein primarily characterised in metabolic cellular processes, as a DNA repair protein further cements the notion of overlapping pathways between metabolism and DNA repair. It has become increasingly evident that these two fundamental cellular processes do not function in isolation and are mutually dependent. ALDOA protein expression is upregulated in many cancers and this upregulation is likely to be critical for the metabolic reprogramming observed in tumours^12–15^.

Although ALDOA did not affect cell proliferation at the timepoints the DNA repair assays were carried out, it’s prolonged depletion did lead to a significant inhibition of cell growth at later timepoints. Together with the observed DNA damage in unperturbed cells, there could be several possibilities for this growth inhibition, including senescence or dependence of the U2OS cell line upon glycolytic metabolism. ALDOA has previously been implicated in senescence in melanoma cell lines^51^, but further investigation in other cancerous and non-cancerous cell lines is required to determine whether senescence is also the cause of the proliferation inhibition observed in the current study.

Here, we have shown an unanticipated role for ALDOA in regulation of the ATM and DNA-PK kinases and subsequently regulation of both DNA DSB repair pathways via HR and NHEJ. Taken together, this suggests that targeting ALDOA may be an important anti-tumour strategy, particularly in tumours that are reliant upon glycolysis as an energy source, and may enable the concurrent targeting of cancer metabolism and DNA repair. This could serve to promote the death of cancer cells following DNA damaging agents, such as IR, commonly used in cancer treatment. Further investigation is required to analyse the feasibility of cancer-targeting strategies that inhibit ALDOA in combination with genotoxic therapies.

### Supplementary Information

Supplementary Information.

## Data Availability

Data and materials are available upon request (E. Bolderson).
