# Supplementary material for: The fructose-bisphosphate, Aldolase A (ALDOA), facilitates DNA-PKcs and ATM kinase activity to regulate DNA double-strand break repair
Source: Sci Rep. 2023 Sep 13;13:15171. doi: 10.1038/s41598-023-41133-1 (PMC10499815; doi:10.1038/s41598-023-41133-1)

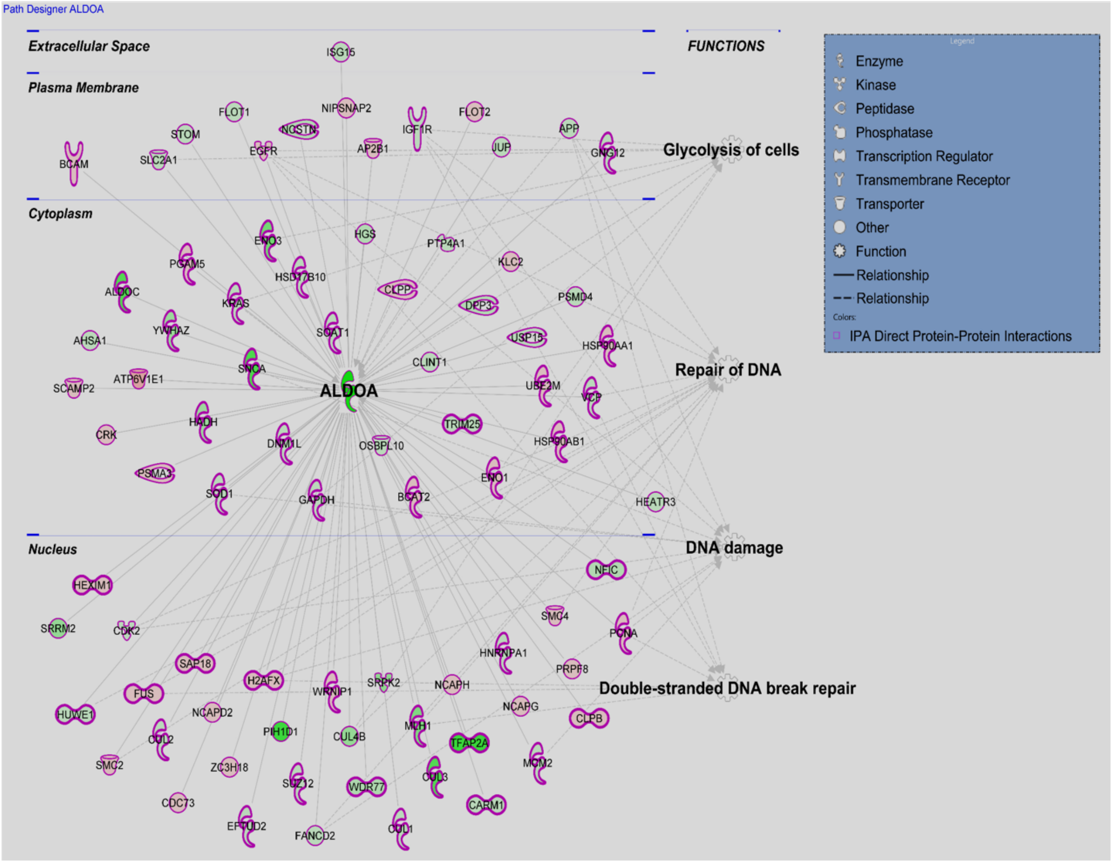

Supplementary Figure 1. Representative network of proteins involved in glycolysis or DNA repair, altered by ALDOA knockdown. Network is annotated from ALDOA 'grown' outwards with all known direct interactors (based on IPA knowledgebase) and annotated with canonical pathways with DNA damage-containing annotations, the layout is based on subcellular locations, from information in IPA. Nodes are limited to proteins present in the proteomics expression dataset. Proteins marked in green are downregulated and red proteins are upregulated.

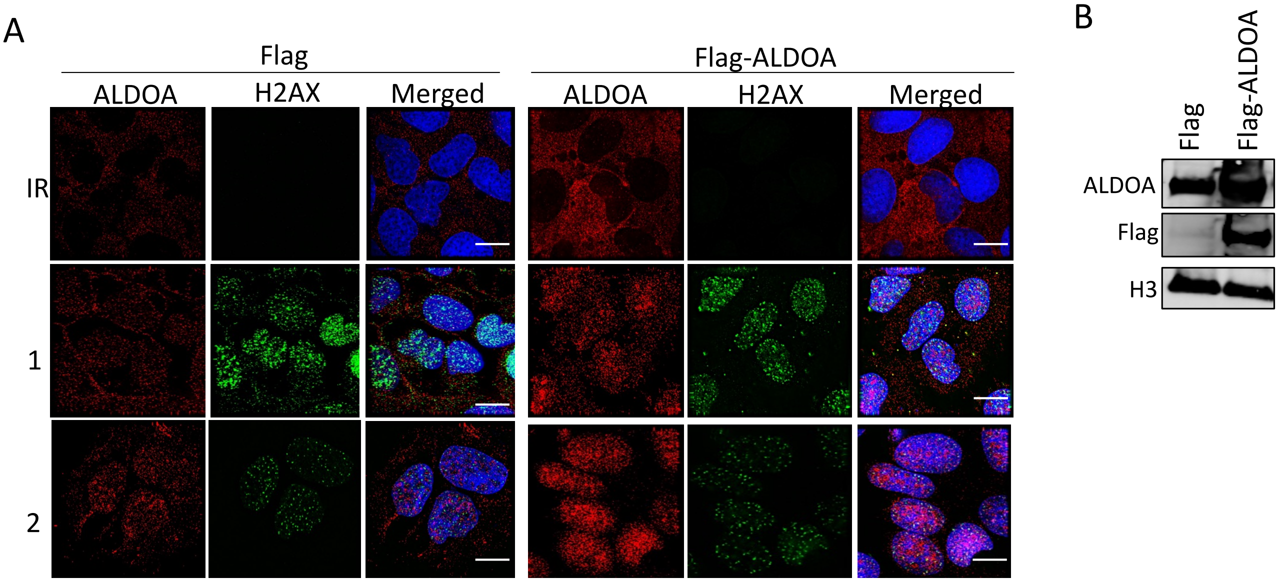

Supplementary Figure 2. ALDOA responds to DNA damage induced by ionising radiation. U2OS cells were transfected with Flag alone or Flag-ALDOA, treated with 6 Gy IR, and pre-extracted and fixed, at the indicated timepoints. Cells were stained with ALDOA and γ-H2AX antibodies and imaged.

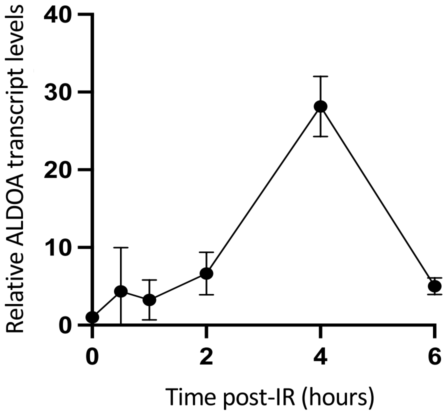

Supplementary Figure 3. ALDOA transcript levels increase after IR. RNA was isolated from U2OS cells at the indicated timepoints following 6 Gy IR. The relative expression of ALDOA mRNA was detected using real-time PCR detected after 6 GY irradiation. Results are representative of 3 independent experiments.

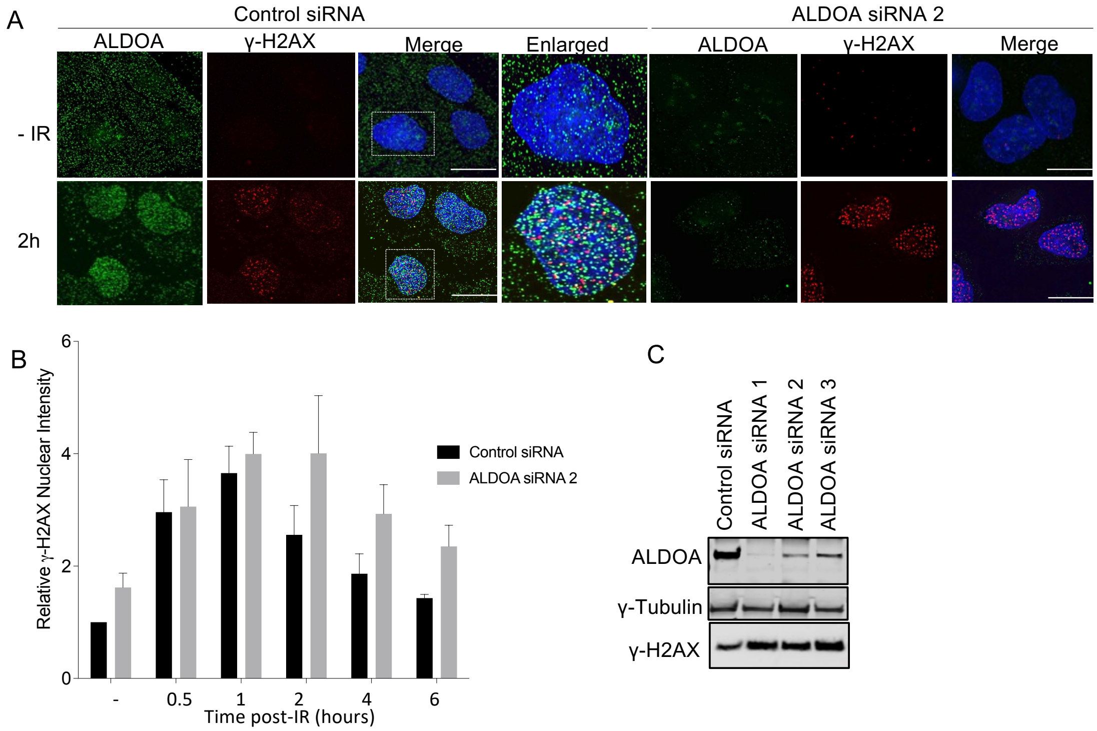

Supplementary Figure 4. Depletion of ALDOA with siRNA 2 leads to slower resolution of histone H2AX phosphorylation. a, U2OS cells were irradiated with 6 Gy of IR. Cells were fixed and immunostained with the indicated antibodies **b**, The histogram represents the the relative nuclear intensity of γ-H2AX from a n=3. **c,** ALDOA knockdown and the effect on γ- H2AX in U2OS cells was determined by Western blot using the indicated antibodies. Images are representative of 3 independent experiments. Immunofluorescence scale bars represent 10 μm.

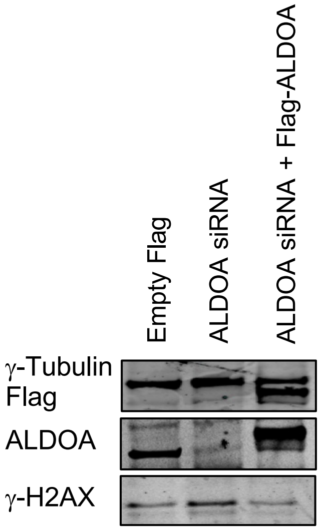

Supplementary Figure 5. Restoration of γ-H2AX levels in U2OS Cell expressing siRNA-resistant Flag-ALDOA (WT). a, Cells were lysed at the indicated times post-IR treatment. SiRNA-resistant Flag-ALDOA expression was determined by Western blot using the indicated antibodies.

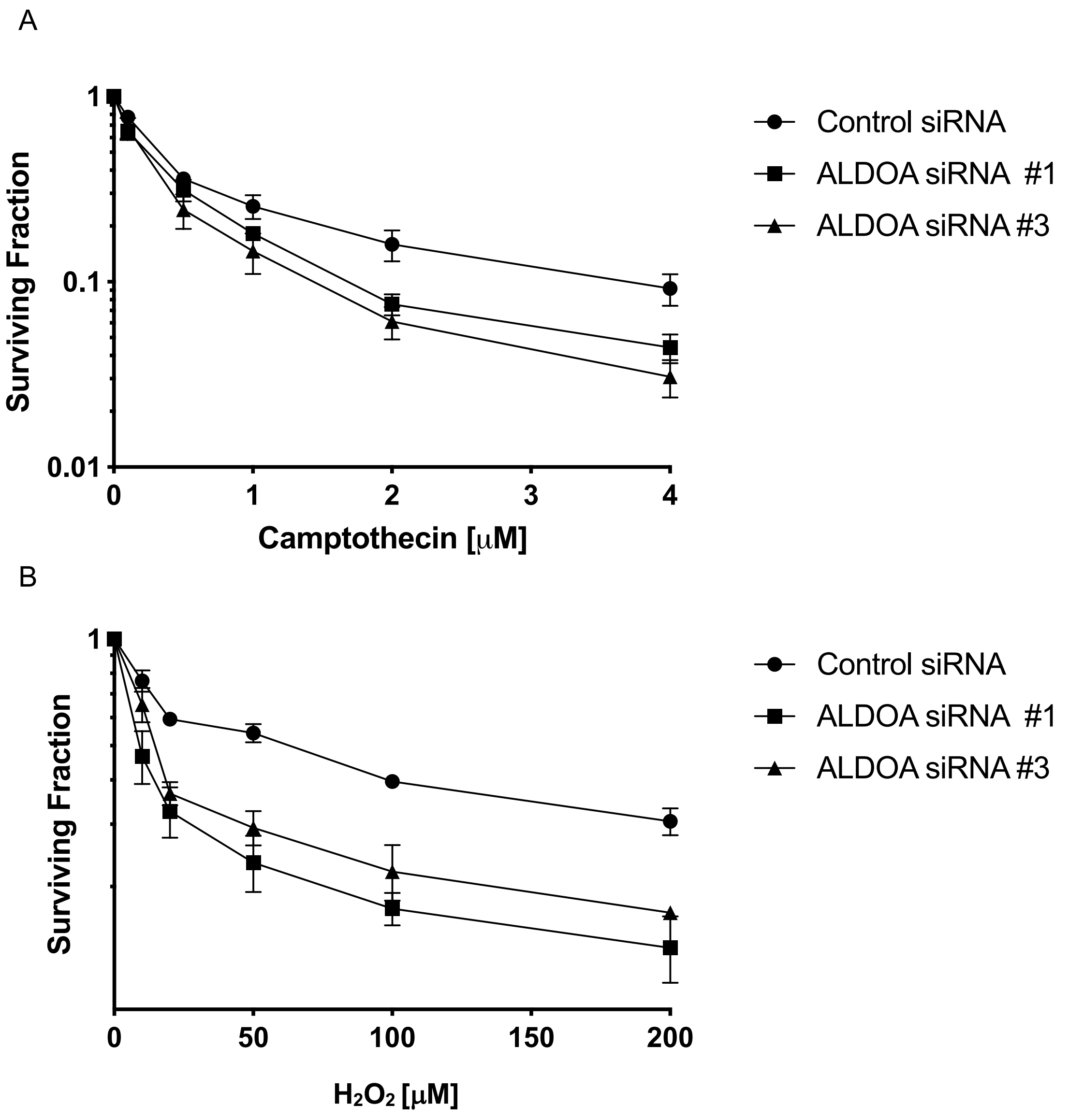

Figure 6. Depletion of ALDOA leads to sensitivity to camptothecin and hydrogen peroxide (H_2O_2). U2OS cells were depleted of ALDOA using siRNA and incubated with the indicated doses of **a,** Camptothecin or **b,** H_2O_2 for 72 hours before the Cell Titre Glo assay was carried out.

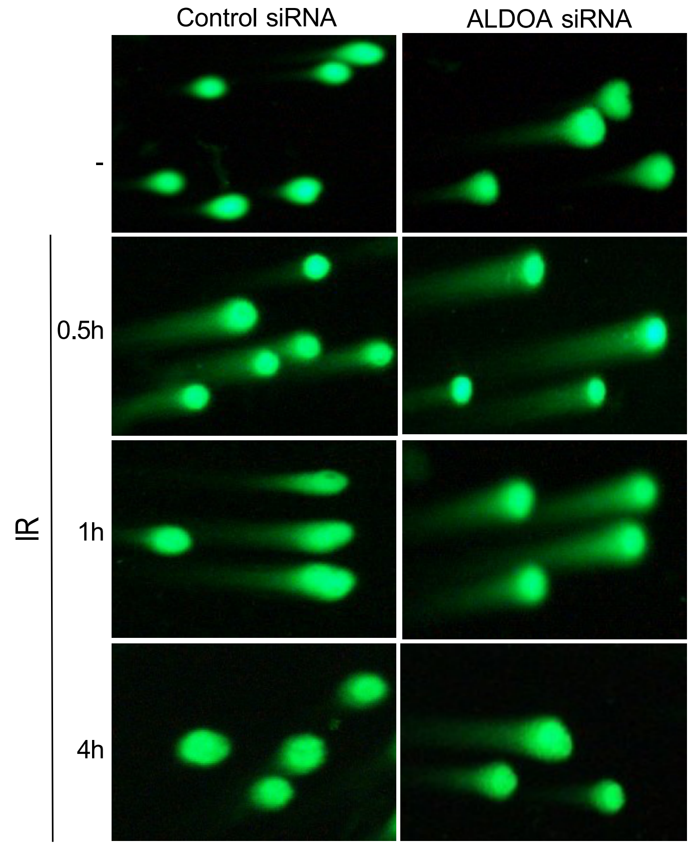

Supplementary Figure 7. Representative comet assay images showing the relative olive tail moment in control or ALDOA siRNA transfected cells, at the indicated times post-IR.

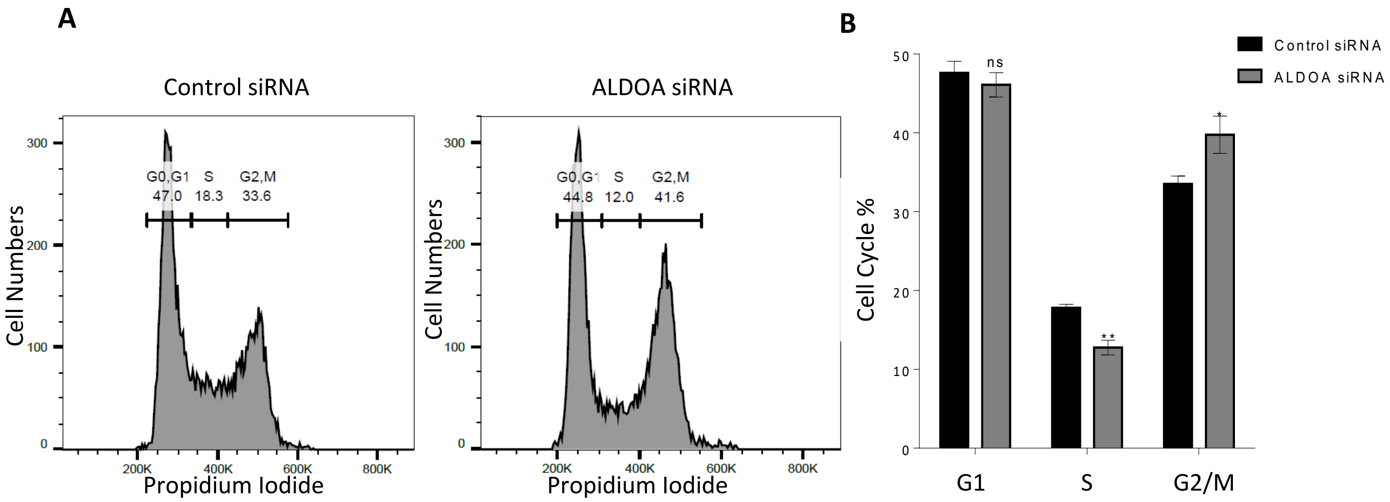

Supplementary Figure 8. The effect of depletion of ALDOA on the cell cycle in U2OS cells. a, Representative cell cycle profiles of control or ALDOA siRNA transfected cells. Histogram data are representative of 3 independent experiments **b**. The percentage of G1, S and G2 cells was calculated from the quadrants. Error bars represent mean ± S.D from three independent experiments.

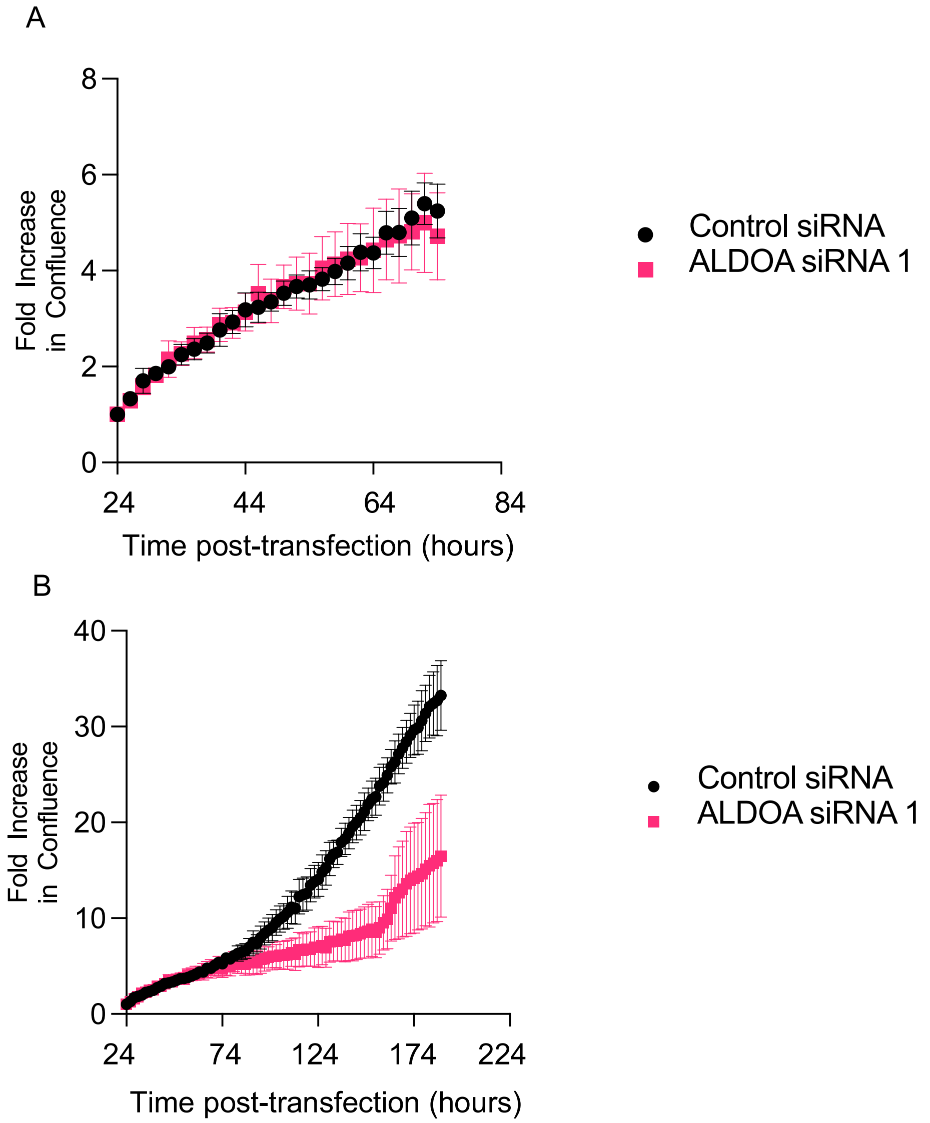

Supplementary Figure 9. Prolonged ALDOA-depletion suppresses the proliferation of U2OS cells. U2OS cells were transfected with control or ALDOA siRNA and analysed on an Incucyte imager from 24 h post-transfection for **a,** 74 h or **b,** 200 h. Data consist of 4 independent repeats and error bars show the standard error of the mean.

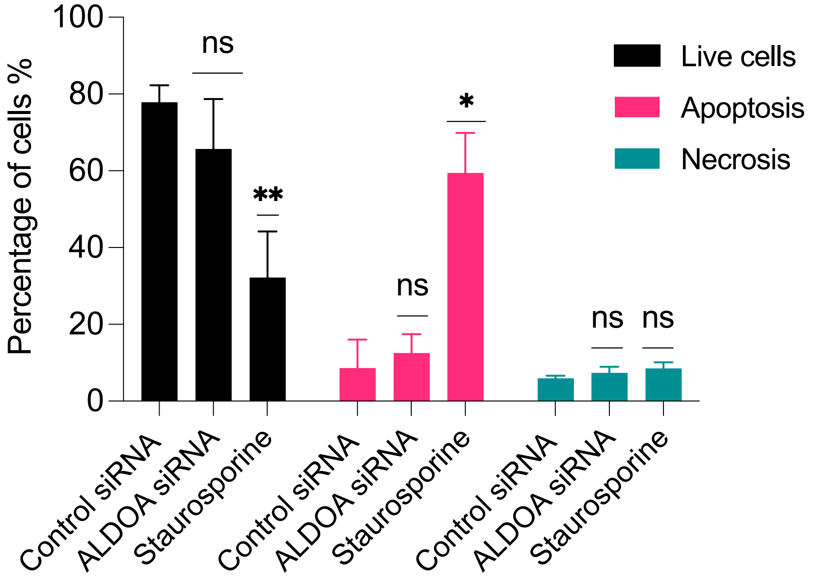

Supplementary Figure 10. ALDOA-depletion does not cause significant cell death at 72 hours post-transfection. U2OS cells were transfected with control or ALDOA siRNA and an Annexin V/PI assay carried out after 72 hours. Staurosporine-treated cells were used as a positive control for apoptosis. Data are representative of 2 independent experiments.

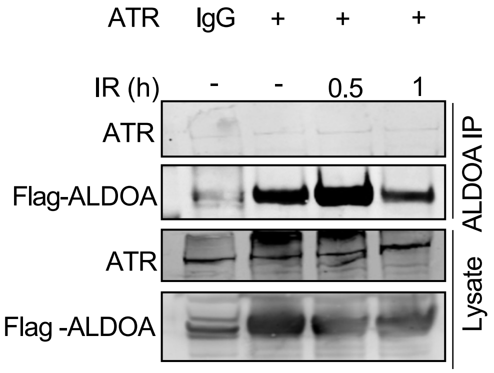

Supplementary Figure 11. ALDOA does not interact with the ATR kinase. Lysates from HEK293T cells expressing Flag or Flag-ALDOA were isolated at the indicated times post-IR. Immunoprecipitates were immunoblotted with the indicated antibodies. Images are representative of 2 independent experiments.

1A

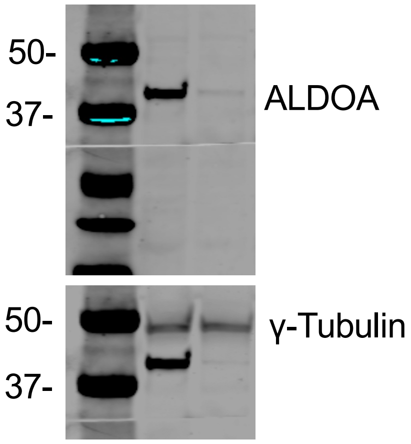

50-
37-
ALDOA
50-
37-
γ-Tubulin

2A

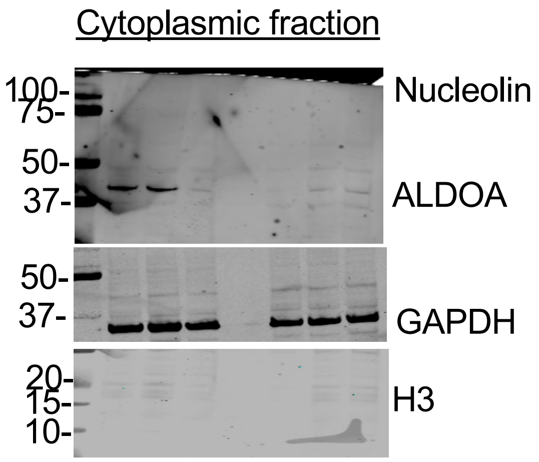

Cytoplasmic fraction
100-
75-
50-
37-
Nucleolin
ALDOA
50-
37-
GAPDH
20-
15-
10-
H3

2A

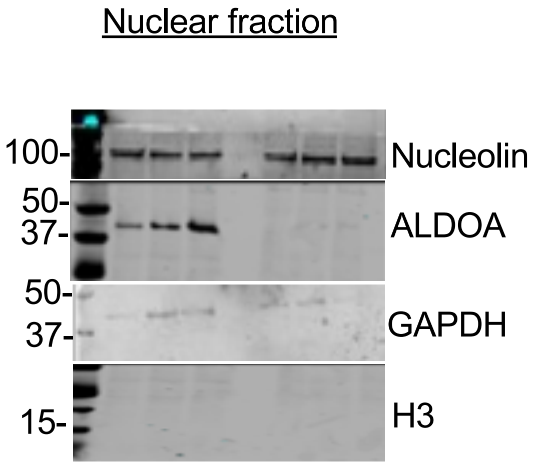

Nuclear fraction
100-
Nucleolin
50-
37-
ALDOA
50-
37-
GAPDH
15-
H3

2A

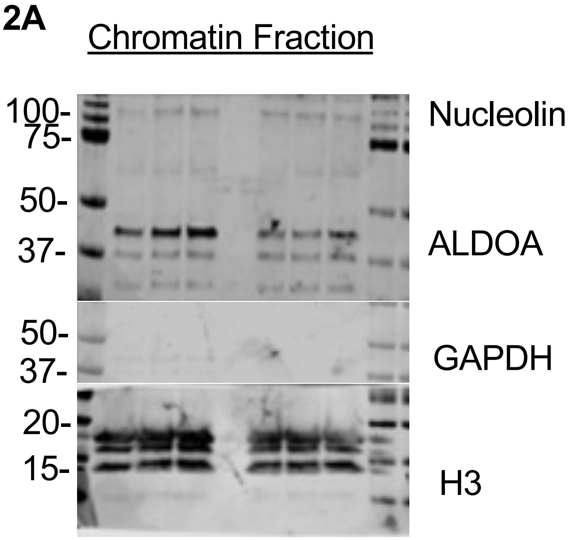

Chromatin Fraction
100-
75-
Nucleolin
50-
37-
ALDOA
50-
37-
GAPDH
20-
15-
H3

2E

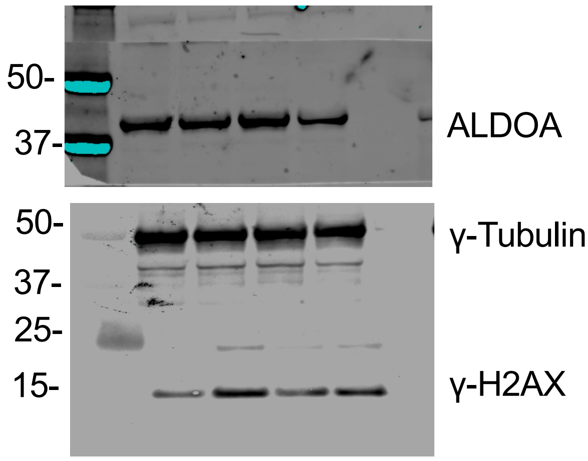

50-
37-
ALDOA
50-
37-
25-
15-
γ-Tubulin
γ-H2AX

4B

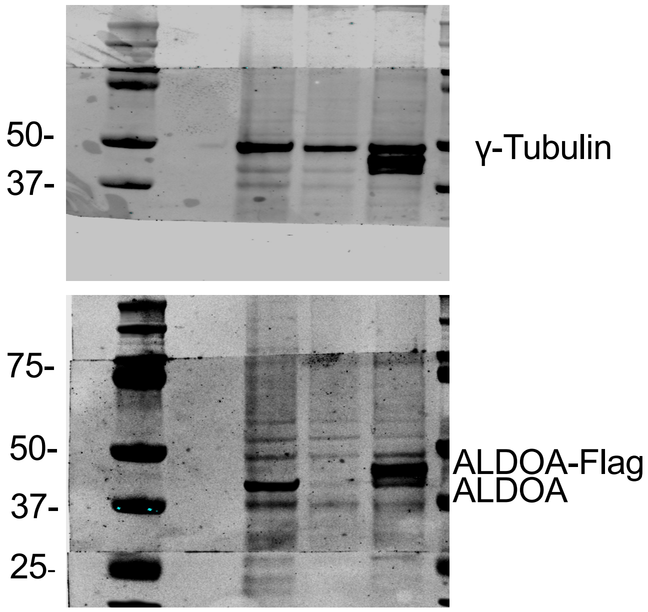
50-
37-
γ-Tubulin
75-
50-
37-
25-
ALDOA-Flag
ALDOA

5A

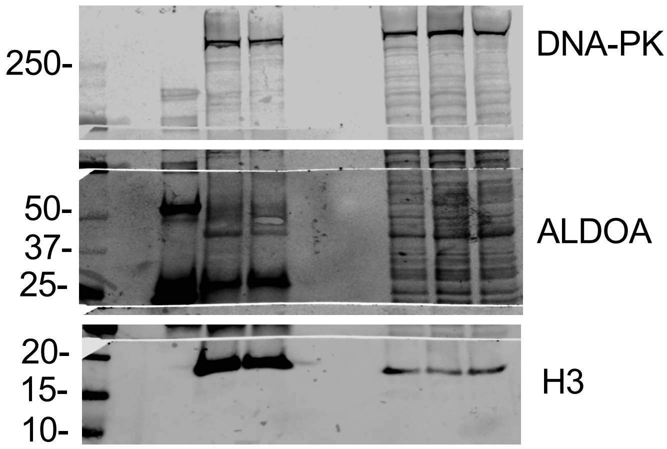
250-
DNA-PK
50-
37-
25-
ALDOA
20-
15-
10-
H3

5B

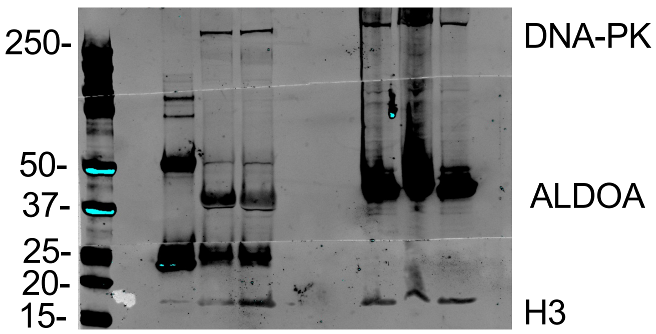
250-
DNA-PK
50-
37-
ALDOA
25-
20-
15-
H3

5C

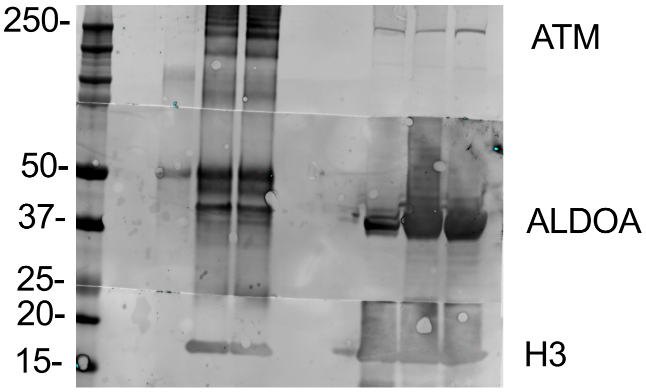
250-
ATM
50-
37-
ALDOA
25-
20-
15-
H3

5D

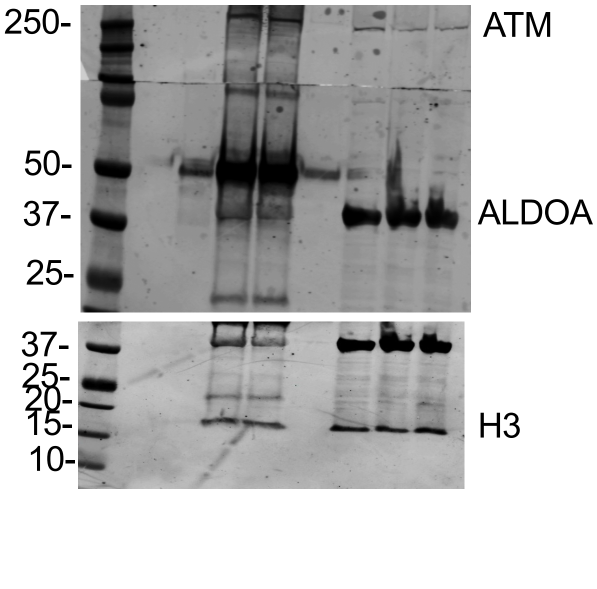
250-
ATM
50-
37-
ALDOA
25-
37-
25-
20-
15-
10-
H3

6A

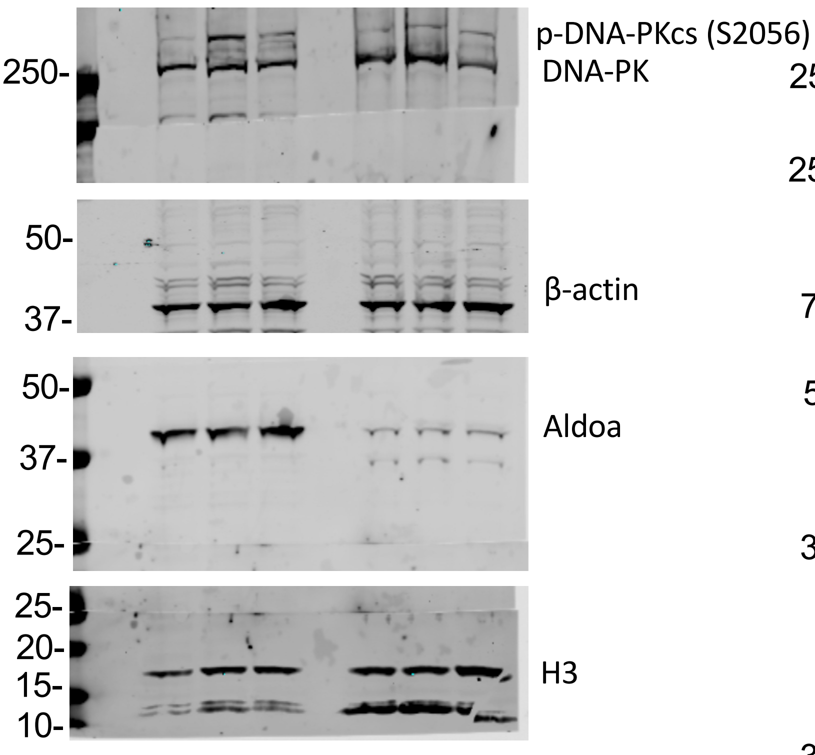
p-DNA-PKcs (S2056)
DNA-PK
250-
50-
37-
β-actin
50-
37-
25-
Aldoa
25-
20-
15-
10-
H3

6B

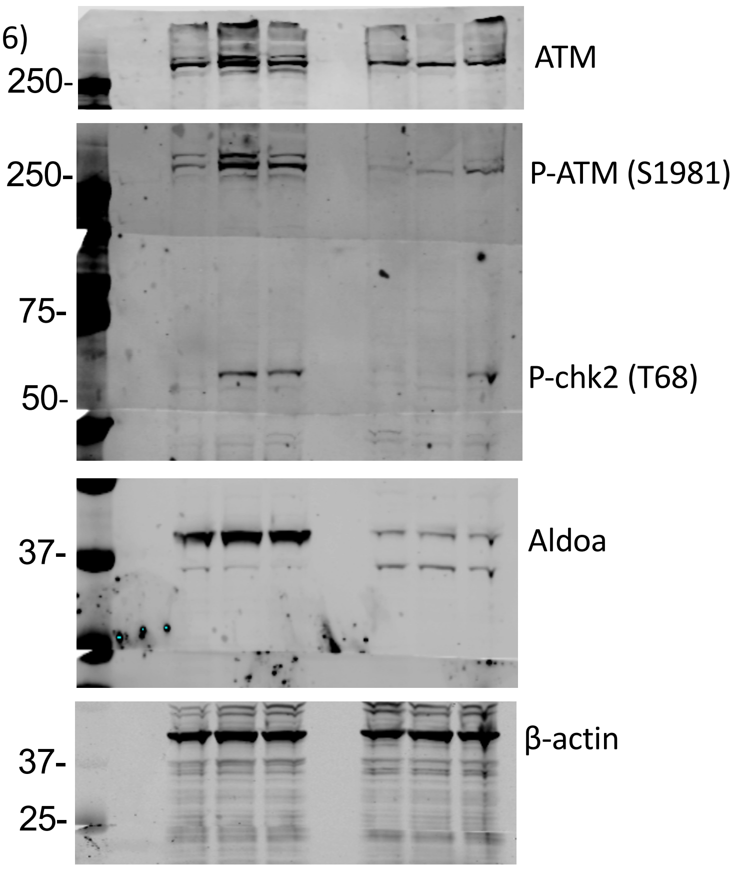
ATM
250-
250-
P-ATM (S1981)
75-
50-
P-chk2 (T68)
37-
Aldoa
37-
25-
β-actin

Supplementary Figures

S2B

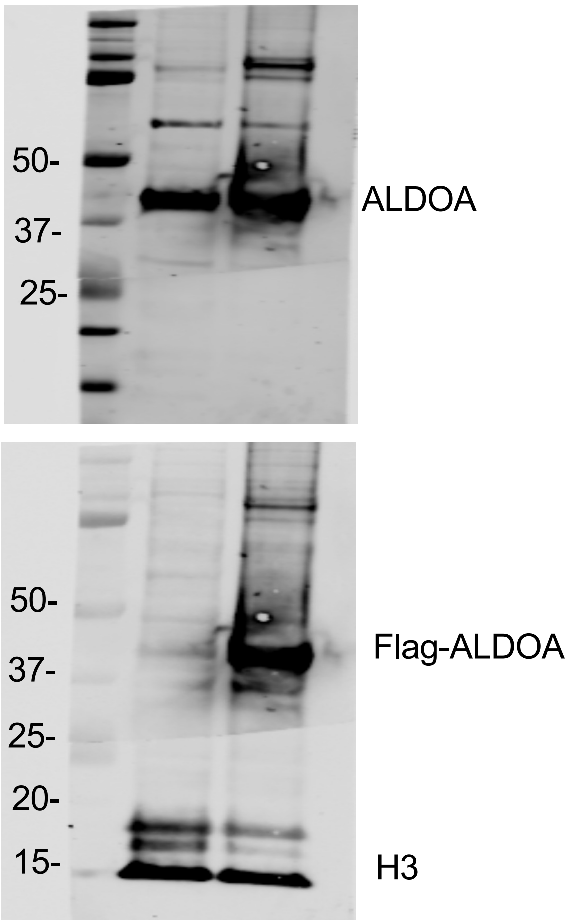

S5

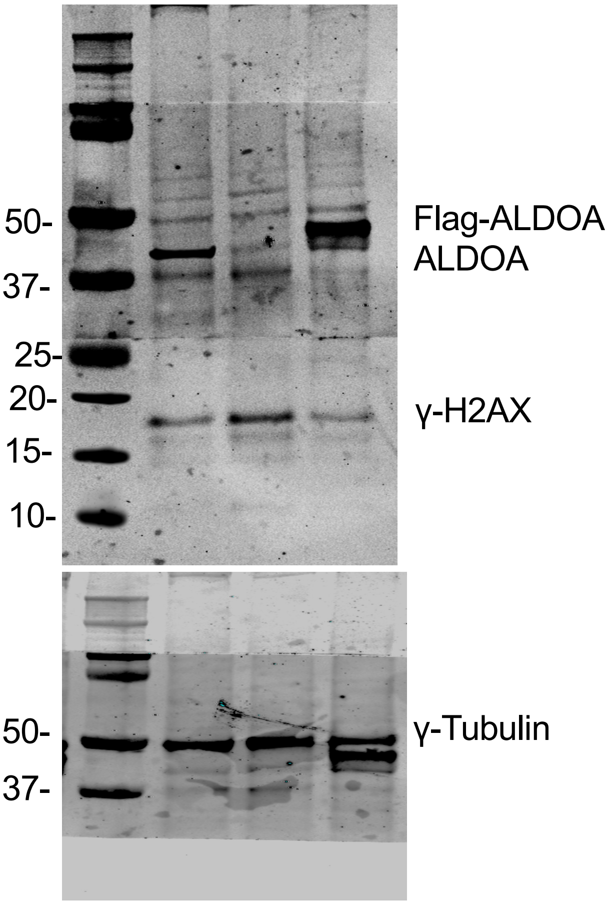

S4C

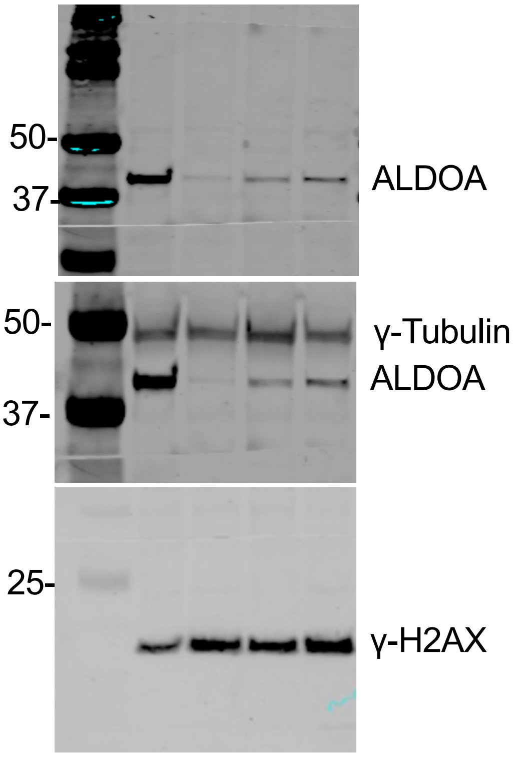

S11

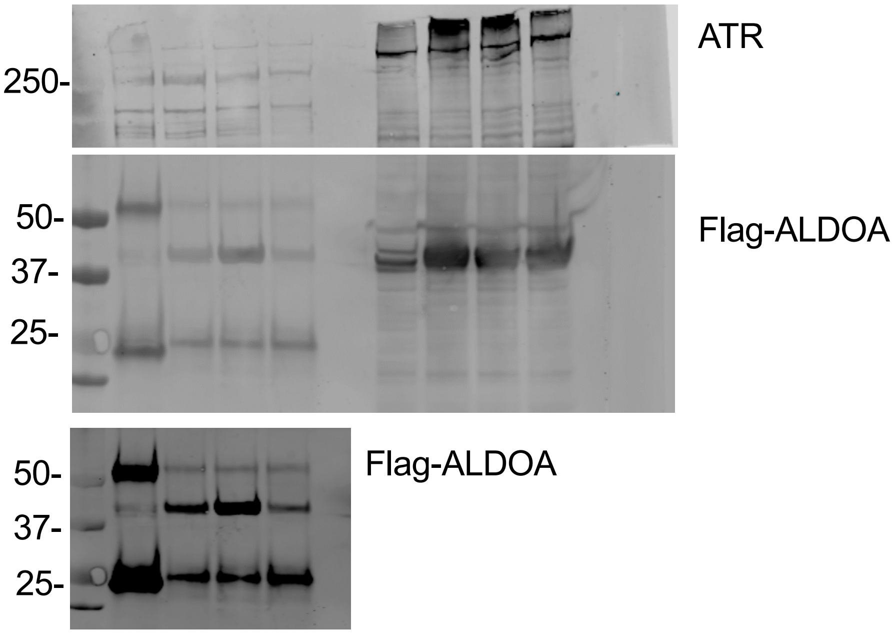

Supplement: Supplementary file 1 — Supplementary Information. [file 41598_2023_41133_MOESM1_ESM.pdf]
